# Lethal abdominal compartment syndrome after extracorporeal cardiopulmonary resuscitation in a patient with out-of-hospital cardiac arrest: a case report

**DOI:** 10.1186/s12245-023-00543-8

**Published:** 2023-09-26

**Authors:** Gun Jik Kim, Kyoung Hoon Lim, Tak-hyuk Oh, Hyun-Joo Lee, Deokbi Hwang, Hanna Jung

**Affiliations:** 1 Department of Thoracic and Cardiovascular Surgery, Kyungpook National University Hospital, Kyungpook National University School of Medicine, Daegu, 41944 Republic of Korea; 2 Department of Surgery, Trauma Center, Kyungpook National University Hospital, Kyungpook National University School of Medicine, Daegu, 41944 Republic of Korea; 3 Department of Thoracic and Cardiovascular Surgery, Kyungpook National University Chilgok Hospital, Kyungpook National University School of Medicine, Daegu, 41404 Republic of Korea; 4 Department of Orthopedic Surgery, Kyungpook National University Hospital, Kyungpook National University School of Medicine, Daegu, 41944 Republic of Korea; 5 Division of Vascular Surgery, Department of Surgery, Kyungpook National University Hospital, Kyungpook National University School of Medicine, Daegu, 41944 Republic of Korea

**Keywords:** Cardiopulmonary resuscitation, Compartment Syndromes, Extracorporeal membrane oxygenation, Fasciotomy, Intensive care units, Laparotomy, Out-of-hospital cardiac arrest

## Abstract

**Background:**

Clinical attempts of extracorporeal cardiopulmonary resuscitation (ECPR) in patients with out-of-hospital cardiac arrest (OHCA) have increased in recent years; however, it also has life-threatening complications. Massive fluid and transfusion resuscitation, shock status, or low cardiac output status during ECPR may lead to ascites and interstitial edema, resulting in secondary abdominal compartment syndrome (ACS).

**Case presentation:**

A 43-year-old male patient was admitted to the emergency department due to cardiac arrest. Due to refractory ventricular fibrillation, ECPR was initiated. Approximately, 3 h after extracorporeal membrane oxygenation support, abdominal distension and rigidity developed. Therefore, ACS was suspected. Decompression laparotomy was required to relieve elevated intra-abdominal pressure.

**Conclusions:**

We report a case of a patient with OHCA who developed lethal ACS after ECPR. Despite this, the patient was able to recover from several major crises. Regardless of how lethal the patient is, if compartment syndrome develops in any part of the body, we should aggressively consider surgical decompression.

## Background

The use of extracorporeal membrane oxygenation (ECMO) during cardiac arrest, also known as extracorporeal cardiopulmonary resuscitation (ECPR), has increased in recent years in patients with inhospital cardiac arrest. Moreover, clinical attempts of ECPR in patients with out-of-hospital cardiac arrest (OHCA) have increased, and these patients are cannulated on arrival in the emergency department [1]. Although ECMO improves the survival and outcome of patients with refractory cardiogenic shock, including cardiac arrest, it also has life-threatening risks and complications. Massive fluid resuscitation, transfusion, shock status, or low cardiac output status during ECPR may lead to ascites and interstitial edema, leading to secondary abdominal compartment syndrome (ACS). Few studies have specifically reported the lethal complications of ACS during ECMO, and unfortunately, there was only one case of survival [2–5]. Here, we report the case of a patient with OHCA who developed lethal ACS after ECPR.

## Case presentation

A 43-year-old male patient was admitted to the emergency department due to cardiac arrest. He recently underwent Holter monitoring due to intermittent palpitations. Multiple premature ventricular contractions were diagnosed and taking beta-blockers. Echocardiography and coronary angiography findings were normal. His electrocardiogram upon arrival at the emergency room showed refractory ventricular fibrillation despite advanced cardiovascular life support algorithms. Therefore, ECPR was initiated. After initiation of ECMO, normal sinus rhythm was recovered. But to maintain adequate blood flow of the ECMO, massive fluid resuscitation (approximately, 1.5L for the first hour and 1L/h for the next 2 hours) with crystalloids was required. 

Prophylactic distal perfusion was placed as soon as admission to intensive care unit. Three hours after ECMO support, abdominal distension and rigidity developed. We attempted nasogastric and rectal tube decompression, which was ineffective. Abdominal rigidity was not released even with the use of a neuromuscular blockade. Enhanced computed tomography revealed bowel edema and no active bleeding. Over time, the abdominal wall became firmer, and bladder pressure was more than 25 mmHg. The patient was presumed to have abdominal compartment syndrome (ACS). The need for decompressive laparotomy was discussed. However, since the patient’s vital signs were barely stable, we decided to observe the patient’s status for a further 24 h.

Eighteen hours after ECMO support, his mean blood pressure was maintained at > 65 mmHg. Transthoracic echocardiography revealed left ventricular dysfunction (left ventricular ejection fraction > 25%). Twenty-four hours after ECMO support, the patient’s right leg showed limb ischemia, even with prophylactic distal perfusion. The arterial cannula needed to be changed or removed from the right common femoral artery. We decided to convert venoarterial (VA) ECMO to venovenous (VV) ECMO. The patient’s vital signs were stable, cardiac rhythm was sinus, and the ejection fraction of the heart was nearly 40% with low-dose inotropic agents after VA ECMO support for 24 h. However, considering the patient’s volume status and lung condition, conversion to VV ECMO support was necessary.

**Fig. 1:**
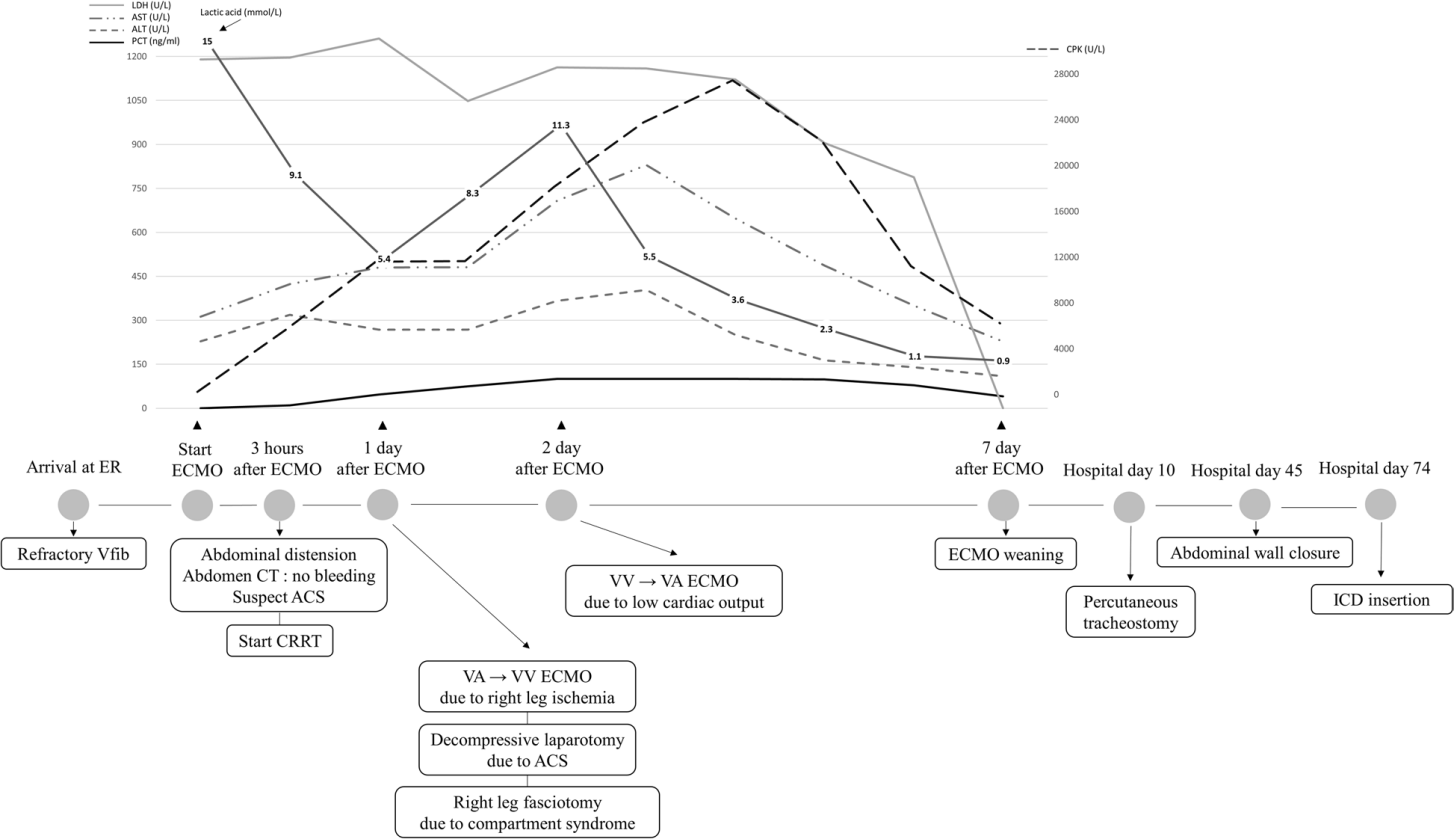
Timeline of the patient treatment and laboratory. ALT, alanine aminotransferase; AST, aspartate aminotransferase; CPK, creatine phosphokinase; CRRT, continuous renal replacement therapy; CT, computed tomography; ER, emergency room; LDH, lactic dehydrogenase; ICD, implantable cardioverter defibrillator; PCT, procalcitonin; Vfib, ventricular fibrillation

Rhabdomyolysis-related laboratory rapidly increased during the 24 h (Fig. 1) after ECMO support without the release of intra-abdominal pressure (IAP). The general surgery team decided to perform decompressive laparotomy. The entire bowel was edematous, and ileum had multiple skipped infarction requiring resection. Although we removed the arterial cannula from the right common femoral artery, the compartment syndrome of the right lower leg progressed, requiring an open fasciotomy of the right lower leg by the orthopedic surgery team.

The patient’s vital signs became unstable under VV ECMO support. Hypotension was refractory to vasoactive agents and volume repletion, and lactic acid increased. We decided to convert VV ECMO to VA ECMO and endure the patient’s low cardiac output. After 24 h of re-supporting VA ECMO, the patient’s vital signs gradually became stabilized. During the next 3 days, inflammatory and rhabdomyolysis-related laboratory slowly improved, and cardiac function was fully recovered (left ventricle ejection fraction > 45%). On the 7th day of admission, ECMO was weaned, and the patient slowly recovered from multi-organ failure. The fasciotomy site on the right lower leg required necrotic muscle resection and skin grafting. Frequent exploration of the bowel and irrigation of the abdominal cavity were performed in the intensive care unit. Right hemicolectomy was needed due to ischemia before the abdominal wall closure. An implantable cardioverter defibrillator was inserted before discharge.

## Discussion

Various studies and detailed guidelines have been reported or updated for decades since the first use of ECMO. However, the survival rate after ECPR for cardiac arrest remains low. The average survival rates for IHCA and OHCA range from 15–17 to 8–10%, respectively [6]. Therefore, numerous reports have recommended indications, inclusion or exclusion criteria, or careful patient selection for favorable outcomes. However, cardiac arrest patients, specifically those with OHCA who are in emergency situations, have a lack of medical information, which makes it difficult to standardize or determine the application of ECPR.

Our patient’s initial electrocardiogram was a shockable rhythm. After ECMO support, the patient’s cardiac rhythm recovered to a normal sinus rhythm. He was just a usually encountering cardiac arrest patient. An OHCA patient is requiring ECPR for refractory ventricular fibrillation. However, 3 h after ECMO support, an unexpected unexperienced complex complication, ACS, developed.

ACS may lead to lethal outcomes if untreated. Our patient was categorized as having secondary ACS due to iatrogenic massive fluid resuscitation to maintain adequate blood flow of ECMO support under life-threatening cardiac arrest. In patients undergoing ECMO support, large-volume fluid administration is frequently required [7, 8]. In our experience, usually 5 to 10 L of volume resuscitation is required in the first 24 h to maintain adequate blood flow for the ECMO support. And initial excessive volume resuscitation in the first 3 to 6 h helps stabilizing the hemodynamic situation of the ECMO support [9]. Patients typically undergo systemic inflammatory response of the ECMO treatment itself which induces pathologic vasodilation and fluid loss to the interstitial compartment, resulting in reduced vascular volume [8]. In addition, massive volume resuscitation, increased capillary permeability secondary to ischemia/reperfusion injury, and/or decreased oncotic pressures can lead to a rapid increase in IAP and eventual ACS [10].

Considering the patient’s lethal status, it was difficult to reach a consensus on the management even through a multidisciplinary team approach. It is known that if IAP aggravates, central venous pressure is increased, and cardiac output is decreased, leading to further ischemic injury and organ malperfusion, a vicious cycle of volume refractory hypoperfusion or hypotension, despite ECMO support. On the other hand, open surgical management, such as decompressive laparotomy, might be too aggressive for the patient since he had disseminated intravascular coagulation and multi-organ failure [7, 10]. Reports have shown poor prognosis with only one successful case report [2, 3, 5].

Our patient underwent decompressive laparotomy 24 h after ACS diagnosis. Rhabdomyolysis-related laboratory rapidly increased, and IAP showed no sign of decrease. The patient could have died from ACS before recovering from cardiogenic shock. Decompressive laparotomy was performed at the bedside of the intensive care unit, which was simpler than expected. Contrary to our expectations, no laparotomy-related bleeding was observed. Once the abdominal wall was opened, the ACS was released, and the patient waited for recovery on ECMO support.

Another educational aspect of this patient was the ECMO support of a critical illness patient suffering severe systemic inflammatory response. ECMO treatment itself and/or multi-organ failure conditions could induce systemic inflammatory response and also could be associated with profound myocardial depression [8, 11]. Our patient initially received VA ECMO for cardiogenic shock caused by refractory ventricular fibrillation. When right leg ischemia became an issue, we converted VA ECMO to VV ECMO instead of changing the VA ECMO cannulation site from the right to the left common femoral artery. At that time, we underestimated the patient’s systemic inflammatory status and only considered the patient’s volume status and lung condition, because the patient’s heart rhythm was stable, and heart function was nearly normal. But actually, the patient was suffering severe systemic inflammation in a critically ill state. We should have waited and supported the cardiac output for the patient to endure or recover from systemic inflammatory status and/or multi-organ failure.

## Conclusions

We report the case of a patient with OHCA who developed lethal ACS after ECPR. Although the patient made a full recovery, the management of the case was based on our knowledge of the condition, which we had not encountered before. Several major crises were encountered even with a multidisciplinary team approach. Nevertheless, management of the case was a great experience. First, regardless of how lethal the condition, if compartment syndrome develops in any part of the body, to help the organ perfusion, surgical decompressing should aggressively considered. Second, if the patient is struggling in a critically ill state, the timing of VA ECMO weaning should be moderately determined. It should be noted that it might be slightly different from weaning VA ECMO in cardiogenic shock, such as in acute myocardial infarction or refractory arrhythmia. There is a need to consider the patient’s entire status and not only the heart function, specifically the need for sufficient cardiac output to endure the critical ill state.

## Data Availability

Not applicable.

## Abbreviations

ACS: Abdominal compartment syndrome
CRP: Cardiopulmonary resuscitation
ECMO: Extracorporeal membrane oxygenation
ECPR: Extracorporeal cardiopulmonary resuscitation
IAP: Intra-abdominal pressure
IHCA: Inhospital cardiac arrest
OHCA: Out-of-hospital cardiac arrest
VA: Venoarterial
VV: Venovenous

## References

[1] MacLaren G, Masoumi A, Brodie D (2020). ECPR for out-of-hospital cardiac arrest: more evidence is needed. Crit Care.

[2] Chang WH (2017). Decompressive laparotomy for abdominal compartment syndrome in patient on extracorporeal life support: a first survival case among adults with literature review. Hong Kong J Emerg Med.

[3] Augustin P, Lasocki S, Dufour G, Rode J, Karsenti A, Al-Attar N (2010). Abdominal compartment syndrome due to extracorporeal membrane oxygenation in adults. Ann Thorac Surg.

[4] Maj G, Calabro MG, Pieri M, Melisurgo G, Zangrillo A, Pappalardo F (2012). Abdominal compartment syndrome during extracorporeal membrane oxygenation. J Cardiothorac Vasc Anesth.

[5] Rollins MD, Deamorim-Filho J, Scaife ER, Hubbard A, Barnhart DC (2013). Decompressive laparotomy for abdominal compartment syndrome in children on ECMO: effect on support and survival. J Pediatr Surg.

[6] Kim H, Cho YH (2020). Role of extracorporeal cardiopulmonary resuscitation in adults. Acute Crit Care.

[7] Hecker A, Hecker B, Hecker M, Riedel JG, Weigand MA, Padberg W (2016). Acute abdominal compartment syndrome: current diagnostic and therapeutic options. Langenbecks Arch Surg.

[8] Kim H, Paek JH, Song JH, Lee H, Jhee JH, Park S (2018). Permissive fluid volume in adult patients undergoing extracorporeal membrane oxygenation treatment. Crit Care.

[9] Djordjevic I, Maier-Trauth J, Gerfer S, Elskamp M, Muehlbauer T, Maul A, et al. Fluid management in veno-arterial extracorporeal membrane oxygenation therapy-analysis of an experimental pig model. J Clin Med. 2023;12(16):5330. 10.3390/jcm12165330 PMC10455548 37629372

[10] Shah SK, Jimenez F, Letourneau PA, Walker PA, Moore-Olufemi SD, Stewart RH (2012). Strategies for modulating the inflammatory response after decompression from abdominal compartment syndrome. Scand J Trauma Resusc Emerg Med.

[11] Al-Fares A, Pettenuzzo T, Del Sorbo L (2019). Extracorporeal life support and systemic inflammation. Intensive Care Med Exp.

